# Ablation of interleukin-19 improves motor function in a mouse model of amyotrophic lateral sclerosis

**DOI:** 10.1186/s13041-021-00785-8

**Published:** 2021-04-30

**Authors:** Hiroyasu Komiya, Hideyuki Takeuchi, Yuki Ogawa, Kosuke Suzuki, Akihiro Ogasawara, Keita Takahashi, Yasu-Taka Azuma, Hiroshi Doi, Fumiaki Tanaka

**Affiliations:** 1 Department of Neurology and Stroke Medicine, Yokohama City University Graduate School of Medicine, 3-9 Fukuura, Kanazawa-ku, Yokohama, 236-0004 Japan; 2 Laboratory of Veterinary Pharmacology, Division of Veterinary Science, Osaka Prefecture University Graduate School of Life and Environmental Science, Izumisano, Osaka 598-9531 Japan

**Keywords:** Amyotrophic lateral sclerosis, Astrocyte, Interleukin-19, Microglia

## Abstract

Neuroinflammation by activated microglia and astrocytes plays a critical role in progression of amyotrophic lateral sclerosis (ALS). Interleukin-19 (IL-19) is a negative-feedback regulator that limits pro-inflammatory responses of microglia in an autocrine and paracrine manner, but it remains unclear how IL-19 contributes to ALS pathogenesis. We investigated the role of IL-19 in ALS using transgenic mice carrying human superoxide dismutase 1 with the G93A mutation (SOD1^G93A^ Tg mice). We generated IL-19–deficient SOD1^G93A^ Tg (IL-19^−/−^/SOD1^G93A^ Tg) mice by crossing SOD1^G93A^ Tg mice with IL-19^−/−^ mice, and then evaluated disease progression, motor function, survival rate, and pathological and biochemical alternations in the resultant mice. In addition, we assessed the effect of IL-19 on glial cells using primary microglia and astrocyte cultures from the embryonic brains of SOD1^G93A^ Tg mice and IL-19^−/−^/SOD1^G93A^ Tg mice. Expression of IL-19 in primary microglia and lumbar spinal cord was higher in SOD1^G93A^ Tg mice than in wild-type mice. Unexpectedly, IL-19^−/−^/SOD1^G93A^ Tg mice exhibited significant improvement of motor function. Ablation of IL-19 in SOD1^G93A^ Tg mice increased expression of both neurotoxic and neuroprotective factors, including tumor necrosis factor-α (TNF-α), IL-1β, glial cell line–derived neurotrophic factor (GDNF), and transforming growth factor β1, in lumbar spinal cord. Primary microglia and astrocytes from IL-19^−/−^/SOD1^G93A^ Tg mice expressed higher levels of TNF-α, resulting in release of GDNF from astrocytes. Inhibition of IL-19 signaling may alleviate ALS symptoms.

## Introduction

Amyotrophic lateral sclerosis (ALS) is an adult-onset neurodegenerative disease characterized by motor neuron degeneration, which leads to progressive muscle weakness, amyotrophy, and death from respiratory paralysis within 3–5 years from onset [1]. ALS is thought to be caused by a multi-factorial mechanism, including glial neuroinflammation, oxidative stress, glutamate-mediated excitotoxicity, mitochondrial abnormalities, and impaired axonal transport [2]. In particular, recent evidence indicates that non-neuronal cells play a pivotal role in ALS pathogenesis [3]. Restricted expression of superoxide dismutase 1 (SOD1) mutation in neurons is not sufficient to induce ALS pathology [4], but wild-type neurons exhibit an ALS phenotype when their surroundings are altered by glial cells carrying the SOD1 mutation [5].

Interleukin-19 (IL-19) is a member of the IL-10 family of cytokines, which includes IL-20, IL-22, IL-26, IL-28A, IL-28B, and IL-29 [6]. Recently, IL-19 was grouped into the IL-20 subfamily, which includes IL-20, IL-22, and IL-24, because these cytokines share common receptor subunits (IL-20 receptor α/β heterodimer) and signaling pathways [7, 8]. IL-19 is an anti-inflammatory cytokine, produced by activated microglia and macrophages, that acts as a negative-feedback regulator to limit pro-inflammatory responses by these cells in an autocrine and paracrine manner [9, 10]. In the central nervous system (CNS), microglia and astrocytes are thought to be the main source of IL-19 production [9, 11]. In regard to its anti-inflammatory role, IL-19 inhibits the symptoms of animal models of inflammatory bowel disease [10, 12], suppresses hapten-dependent skin inflammation in contact hypersensitivity [13], decreases brain infarction area in a mouse model of cerebral ischemia [14], and improves locomotor function in a mouse model of spinal cord injury [15]. On the other hand, IL-19 also acts as a pro-inflammatory cytokine in T helper 2 cell (Th2)-mediated diseases such as asthma and rheumatoid arthritis. In fact, IL-19 stimulation induces the production of TNF-α, IL-6, and reactive oxygen species in monocytes [16], TNF-α and IL-6 in synovial fibroblasts [17], and IL-1β, IL-6, IL-8, CCL5, and CXCL9 in lung epithelial cells [18]. Therefore, IL-19 exerts either anti-inflammatory or pro-inflammatory effects in accordance with immunological conditions.

However, it remains uncertain whether IL-19 is involved in the pathomechanism of neurodegenerative diseases. In this study, we used a mouse model to investigate how IL-19 contributes to the pathogenesis of ALS.

## Methods

### Animals

All animal experiments were conducted under protocols approved by the Animal Experiment Committee of Yokohama City University (approved number: F-A-19–036). C57BL/6 (B6) mice were purchased from Japan SLC (Hamamatsu, Japan). IL-19^−/−^ mice (B6 background) were obtained from Regeneron Pharmaceuticals (Tarrytown, NY, USA) [10]. Transgenic mice carrying 23 copies of a transgene encoding the G93A mutant of human SOD1 (designated as SOD1^G93A^ Tg mice) were purchased from the Jackson Laboratory (B6.Cg-Tg(SOD1-G93A)1Gur/J; #004435, Jackson Laboratory, Bar Harbor, ME, USA) [19]. We obtained IL-19–deficient SOD1^G93A^ Tg mice (designated as IL-19^−/−^/SOD1^G93A^ Tg mice) by crossing SOD1^G93A^ Tg mice with IL-19^−/−^ mice.

### Behavioral assessments

After the age of 8 weeks, mice were assessed weekly by body weight scaling and Rotarod test; assessments were performed in randomized order by an observer blinded to the genotype [20]. Time of disease onset was defined as the first day when the mice exhibited hindlimb motor deficits such as abnormal reflexes or tremor, which are early symptoms observed in SOD1^G93A^ Tg mice [20, 21]. For the Rotarod test, the time that the mouse remained on a rotating cylinder (Ugo-Basile, Monvalle, Varese, Italy) at a constant speed of 15 rpm was measured. Each mouse was evaluated three times, and the longest latency to fall was recorded; 180 s was chosen as the arbitrary cut-off time.

### Cells

Primary cultures were prepared from SOD1^G93A^ Tg mice or IL-19^−/−^/SOD1^G93A^ Tg mice. Primary microglia cultures were isolated from primary mixed glial-cell cultures prepared from newborn mice using the “shaking off” method after 14 days in vitro, as described previously [3, 22]. The purity of the cultures, as determined by anti-CD11b immunostaining, was > 99%. Cultures were maintained in Dulbecco’s modified Eagle’s minimum essential medium (DMEM) (Sigma-Aldrich, St. Louis, MO, USA) supplemented with 10% fetal bovine serum (FBS) (Equitech-Bio, Kerrville, TX, USA), 5 μg/ml bovine insulin (Sigma-Aldrich), and 0.2% glucose. Primary astrocyte cultures were purified from primary mixed glial cultures by three to four repetitions of trypsinization and replating, as described previously [4, 23]. Astrocyte purity, as determined by glial fibrillary acidic protein (GFAP)-specific immunostaining, was > 95%.

### Quantitative reverse transcription PCR analysis

Lumbar spinal cords were collected from 20-week-old mice. Microglia and astrocytes were seeded at a density of 1 × 10^5^ cells/well (500 µl/well) in 48-well plates. Microglia were treated with 0–1000 ng/ml lipopolysaccharide (LPS) for 24 h. Astrocytes were treated with 0–1000 ng/ml LPS for 24 h. Total RNA was extracted from cultured microglia, astrocytes, and lumbar spinal cords using the miRNeasy Mini Kit (Qiagen, Valencia, CA, USA). For quantitative reverse transcription PCR (qPCR) experiments, RNA was reverse transcribed into cDNA using SuperScript III (Life Technologies, Carlsbad, CA, USA). Levels of mRNAs encoding IL-19, IL-6, IL-10, inducible nitric oxide synthase (iNOS), tumor necrosis factor α (TNF-α), IL-1β, glial cell line–derived neurotrophic factor (GDNF), brain-derived neurotrophic factor (BDNF), TGF-β1, IL-20 receptor α (IL-20Rα), IL-20 receptor β (IL-20Rβ), and hypoxanthine phosphoribosyltransferase 1 (HPRT1) were measured using quantitative PCR (qPCR), which was performed on a LightCycler using the KAPA SYBR FAST qPCR Master Mix kit (Sigma-Aldrich). Relative expression levels were determined using the ΔΔC_T_ method; the genes of interest were normalized against the geometric mean of HPRT1. Primers for qPCR are indicated in Table1.

**Table 1:** Primers for qPCR

Gene	Primer sequence
mouse *Il19* sense	TACAGAGACAGGGTGTTCCAGGAC
mouse *Il19* antisense	GCATTGGTGGCTTCCTGACTGCAGT
mouse *Il6* sense	ACAAGTCGGAGGCTTAATTACACAT
mouse *Il6* antisense	AATCAGAATTGCCATTGCACAA
mouse *Il10* sense	ACAAGTCGGAGGCTTAATTACACAT
mouse *Il10* antisense	AATCAGAATTGCCATTGCACAA
mouse *Nos2* sense	CAGCTGGGCTGTACAAACCTT
mouse *Nos2* antisense	GGGATCTGAATGTGATGTTTGGCT
mouse *Il1b* sense	GAAATGCCACCTTTTGACAGTG
mouse *Il1b* antisense	TGGATGCTCTCATCAGGACAG
mouse *Tnfa* sense	GACCCTCACACTCAGATCATCTTCT
mouse *Tnfa* antisense	CCACTTGGTGGTTTGCTACGA
mouse *Tgfb1* sense	CGAAGCGGACTACTATGCTAAAGA
mouse *Tgfb1* antisense	GTTTTCTCATAGATGGCGTTGTTG
mouse *Gdnf* sense	CGGACGGGATAAGATGA
mouse *Gdnf* antisense	CTGCCGCTTGTTTATCTGGT
mouse *Bdnf* sense	GATGCCGCAAACATGTCTATGA
mouse *Bdnf* antisense	TAATACTGTCACACACGCTCAGCTC
mouse *Il20ra* sense	GGAAACTCAAGTCAGCCCAC
mouse *Il20ra* antisense	AGATGGACTTCTCGCCAGTT
mouse *Il20rb* sense	CCGAAATGCAACTGTCCTCAC
mouse *Il20rb* antisense	AATAACCAGATGCAGCCCATGT
mouse *Hprt1* sense	TCAGTCAACGGGGGACATAAA
mouse *Hprt1* antisense	CTGGTTAAGCAGTACAGCCCC

### Histological analysis

Frozen sections (20 µm thick) of mouse lumbar spinal cords were prepared using a previously described method [20, 24] at each disease stage (early stage, 12 weeks; middle stage, 16 weeks; late stage, 20 weeks; end stage, 24 weeks). The sections were permeabilized with 1% Triton-X-100 in PBS for 30 min, blocked with 10% FBS for 1 h, and incubated overnight with rabbit anti–mouse Iba1 polyclonal antibodies (1:300; Wako, Tokyo, Japan), mouse anti-NeuN monoclonal antibody (CI: A60, 1:500; Chemicon, Temecula, CA, USA), rabbit anti–mouse GFAP polyclonal antibodies (1:1000; Dako, Glostrup, Denmark), and rat anti-CD68 monoclonal antibody (CI: FA-11, 1:500; Bio-Rad, Hercules, CA, USA), followed by incubation with Alexa Fluor 488 or Alexa Fluor 546–conjugated secondary antibodies (Life Technologies). The stained cells were analyzed in six random fields per section using a deconvolution fluorescence microscope system (BZ-X800; Keyence, Osaka, Japan) as described previously [20, 24]. Data were collected from six sections per mouse and four mice per group.

### Statistical analysis

Statistical significance was analyzed using Student’s *t*-test or one-way analysis of variance (ANOVA) followed by post hoc Tukey’s test. Survival time and onset time were analyzed by log-rank test. All statistical analyses were performed using GraphPad Prism version 8 (Graph Pad Software, La Jolla, CA, USA).

## Results

### IL-19 is upregulated in primary microglia and lumbar spinal cord of SOD1^G93A^ Tg mice

**Fig. 1:**
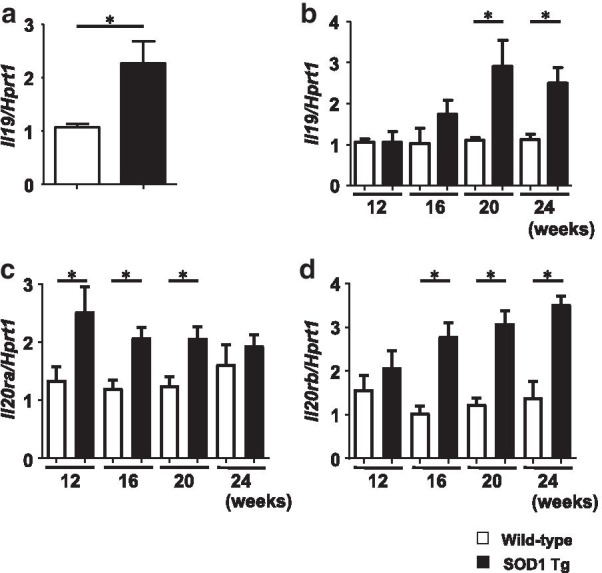
Expression levels of IL-19 and IL-19 receptor are elevated in primary microglia and lumbar spinal cord of SOD1Tg mice. **a** qPCR data for IL-19 in primary microglia of wild-type and SOD1^G93A^Tg mice. **b** qPCR data for IL-19 in lumbar spinal cord of wild-type and SOD1^G93A^Tg mice. **c** qPCR data for IL-20Rα in lumbar spinal cord of wild-type and SOD1^G93A^Tg mice. **d** qPCR data for IL-20Rβ in lumbar spinal cord of wild-type and SOD1^G93A^Tg mice. Values are means ± SD (n = 4). *p < 0.05

First, we examined IL-19 expression levels in primary microglia and lumbar spinal cords of wild-type and SOD1^G93A^ Tg mice. qPCR analyses revealed that IL-19 expression in primary microglia was significantly higher in SOD1^G93A^ Tg mice than in wild-type mice (Fig. 1a). SOD1^G93A^ Tg mice also exhibited elevated expression of IL-19 in the lumbar spinal cords as the disease progressed (Fig. 1b). In addition, expression levels of IL-19 receptor (IL-20 receptor α/β heterodimer) were higher in lumbar spinal cords of SOD1^G93A^ Tg mice than in wild-type mice. (Fig. 1c, d).

### Ablation of IL-19 in SOD1^G93A^ Tg mice improves motor function, but not lifespan

**Fig. 2:**
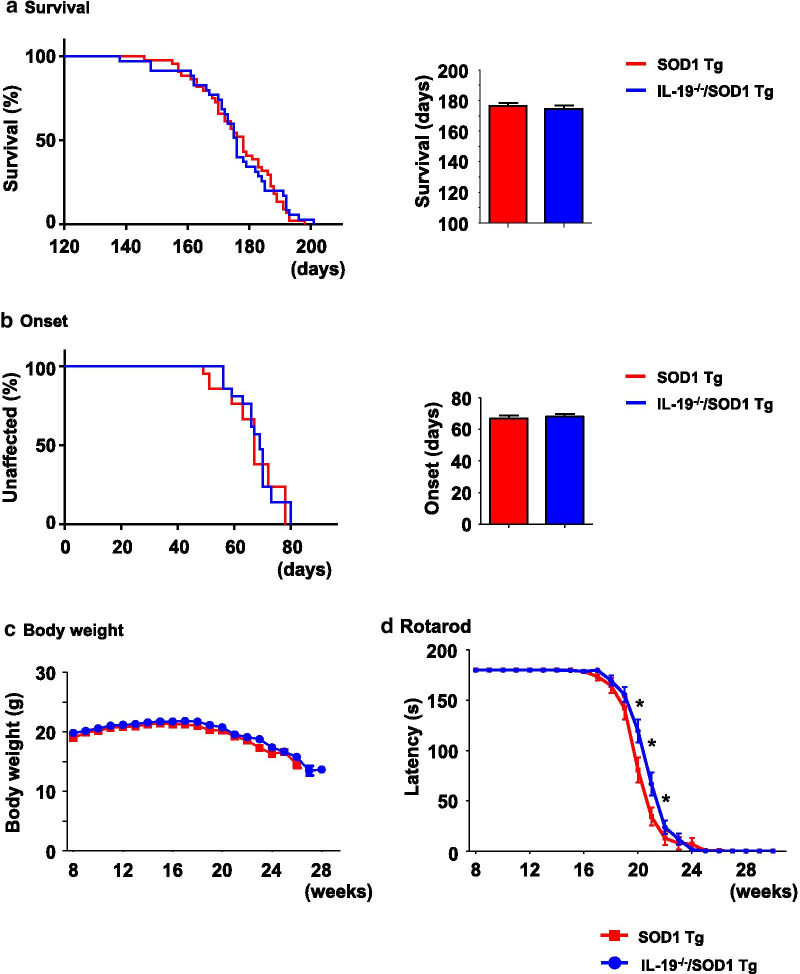
Interleukin-19 deficiency improves motor function at the late stage in the SOD1^G93A^Tg mice. **a** Kaplan–Meier survival curves for SOD1^G93A^Tg and IL-19^−/−^/SOD1^G93A^Tg mice. **b** Kaplan–Meier curves for time to onset of SOD1^G93A^Tg and IL-19^−/−^/SOD1^G93A^Tg mice. **c** Body weight. Significant differences were not observed between the two types of model mice. **d** Rotarod test. IL-19^−/−^/SOD1^G93A^Tg mice exhibited a significant improvement in motor function at the late stage (20–22 weeks) relative to SOD1^G93A^Tg mice. Values are means ± SD (SOD1^G93A^Tg, n = 29; IL-19^−/−^/SOD1^G93A^Tg, n = 30). *, *p* < 0.05

Next, we investigated the role of IL-19 in ALS by comparing SOD1^G93A^ Tg mice with IL-19^−/−^/SOD1^G93A^ Tg mice. IL-19 deficiency did not cause a significant difference in mean survival time (Fig. 2a; IL-19^−/−^/ SOD1^G93A^ Tg, 178.4 ± 11 days *vs.* SOD1^G93A^ Tg, 173.6 ± 12 days), disease onset (Fig. 2b; IL-19^−/−^/ SOD1^G93A^ Tg, 68.1 ± 1.6 days *vs.* SOD1^G93A^ Tg, 66.8 ± 2.0 days), or body weight (Fig. 2c), but it did significantly improve motor function at the late stage of the disease (Fig. 2d, 20–22 weeks). These results indicated that IL-19 influences disease progression in SOD1^G93A^ Tg mice.

### Ablation of IL-19 in SOD1^G93A^ Tg mice alters microglial phenotype in lumbar spinal cords

**Fig. 3:**
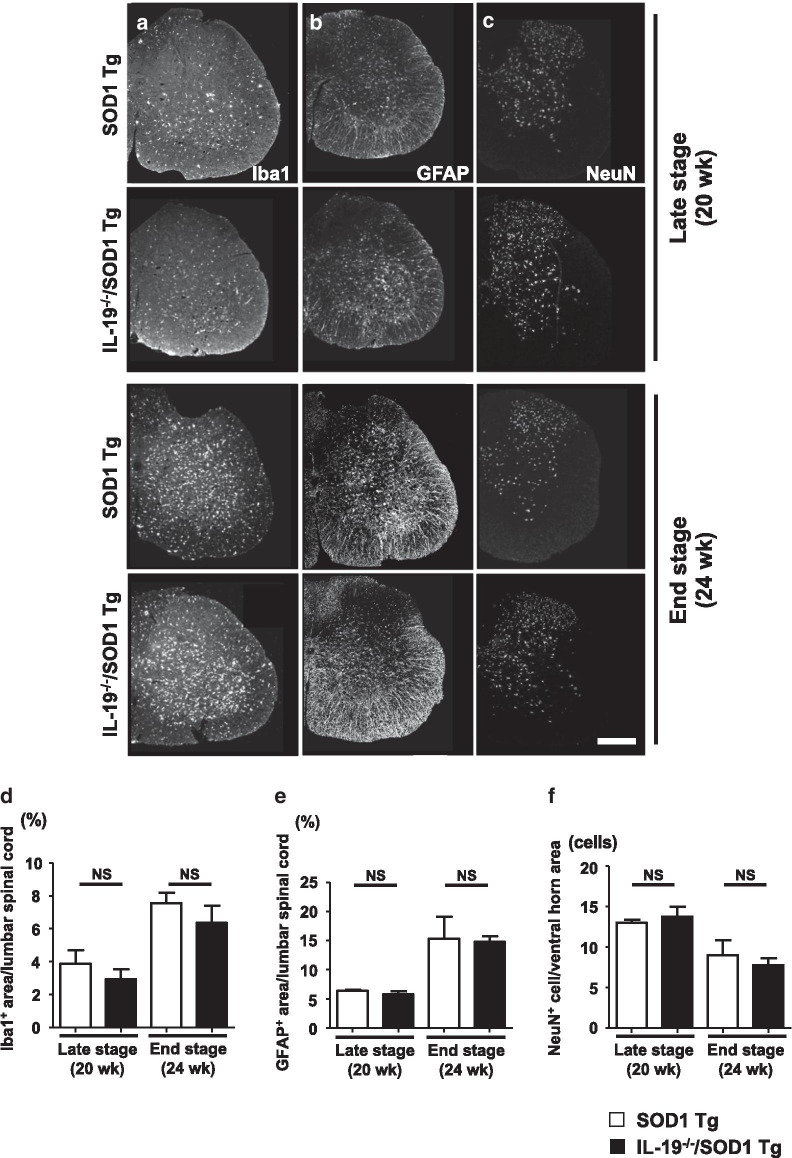
Ablation of IL-19 does not affect gliosis and neuronal loss in SOD1^G93A^ Tg. **a**–**c** Images of lumbar spinal cord of SOD1^G93A^ Tg and IL-19^−/−^/ SOD1^G93A^ Tg mice at the late stage (20 weeks) and end stage (24 weeks), immunostained for (**a**) Iba1, (**b**) GFAP, and (**c**) NeuN. **d** Percentage of Iba1-positive areas in the lumbar spinal cord sections. **e** Percentage of GFAP-positive areas in the lumbar spinal cord sections. **f** Numbers of NeuN-positive cells in the ventral horn of lumbar spinal cord sections. Scale bar, 200 µm. Values are means ± SD

**Fig. 4:**
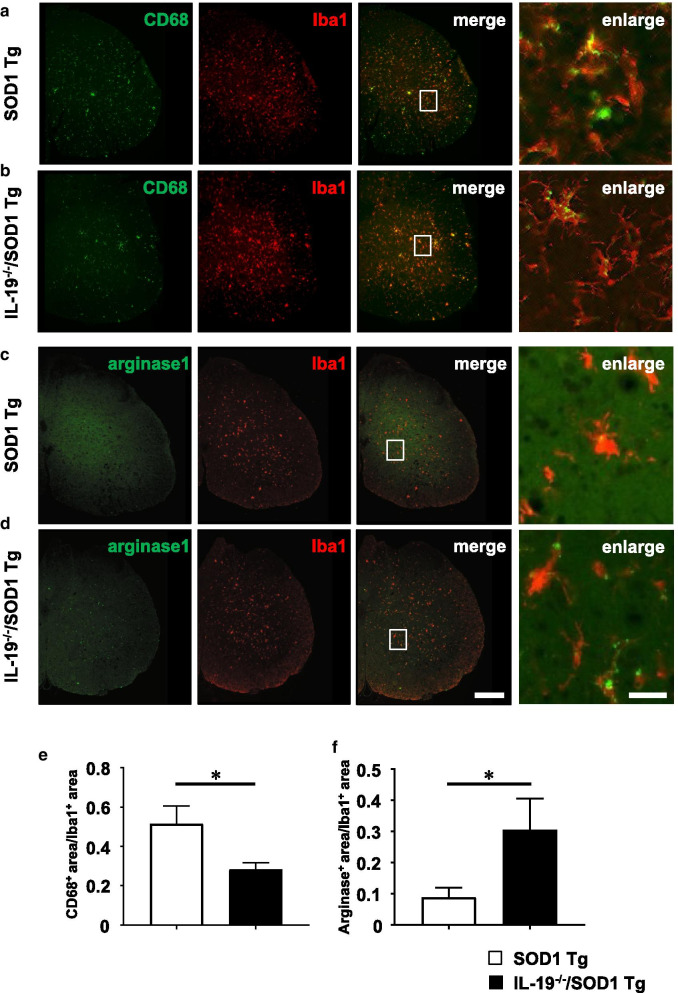
Ablation of IL-19 modulates microglial phenotype in SOD1^G93A^ Tg at the late stage. Images of lumbar spinal cord of 20-week-old (**a**) SOD1^G93A^Tg mice and (**b**) IL-19^−/−^/SOD1^G93A^ Tg mice immunostained for CD68 (green) and Iba1 (red). Images of lumbar spinal cord of 20-week-old (**c**) SOD1^G93A^ Tg mice and (**d**) IL-19^−/−^/SOD1^G93A^ Tg mice immunostained for arginase 1 (green) and Iba1 (red). **e** Percentage of CD68 positive cells in Iba1 positive cells in lumbar spinal cord sections. **f** Percentage of arginase 1 positive cells in Iba1 positive cells in the lumbar spinal cord sections. Scale bars: 200 µm in merged images and 50 µm in enlarged images. Values are means ± SD. *p < 0.05

Next, we examined the effect of IL-19 on glial cells in lumbar spinal cord at the late (20 weeks) and end (24 weeks) stages. Immunohistochemical analyses of microglia, astrocytes, and neurons did not reveal significant differences in the extent of gliosis or neuronal loss between SOD1^G93A^ Tg mice and IL-19^−/−^/SOD1^G93A^ Tg mice at either disease stage (Fig. 3a–f). However, ablation of IL-19 decreased positivity for CD68 (neurotoxic microglial marker; Fig. 4a, b, e) and increased positivity for arginase1 (neuroprotective microglial marker; Fig. 4c, d, f) in lumbar spinal cord microglia of 20-week-old SOD1^G93A^Tg mice. These data indicated that IL-19 modulates microglial phenotype in SOD1^G93A^ Tg mice.

### Ablation of IL-19 upregulates both neurotoxic and neuroprotective factors in lumbar spinal cord of SOD1^G93A^ Tg mice

**Fig. 5:**
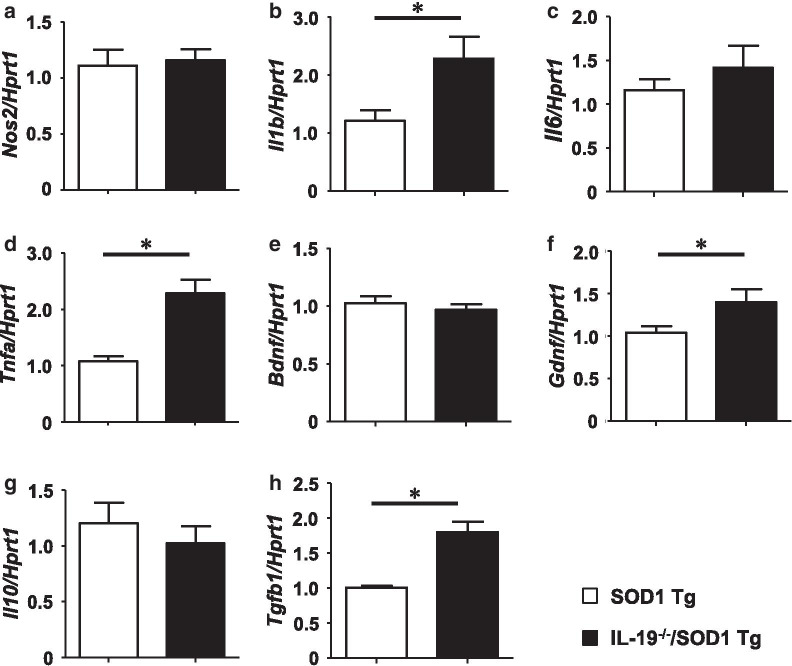
qPCR analyses of pro-inflammatory cytokines, anti-inflammatory cytokines, and growth factors in the lumbar spinal cords of 20-week-old SOD1^G93A^ Tg mice and IL-19^−/−^/ SOD1^G93A^ Tg mice. qPCR data are shown for **a** iNOS mRNA, **b** IL-1β mRNA, **c** IL-6 mRNA, **d** TNF-α mRNA, **e** BDNF mRNA, **f** GDNF mRNA, **g** IL-10 mRNA, and **h** TGF-β1 mRNA. Values are means ± SD (n = 4). *p < 0.05

Next, we evaluated the expression levels of neurotoxic and neuroprotective factors in the lumbar spinal cords of SOD1^G93A^ Tg and IL-19^−/−^/SOD1^G93A^ Tg mice at the late stage (20 weeks) when motor function began to show a significant difference. qPCR analyses for pro-inflammatory factors revealed that expression levels of TNF-α and IL-1β were significantly upregulated in IL-19^−/−^/SOD1^G93A^ Tg mice compared to SOD1^G93A^ Tg mice (Fig. 5a–d). As for neurotrophic factors and anti-inflammatory factors, GDNF and TGF-β were significantly upregulated in IL-19^−/−^/SOD1^G93A^ Tg mice compared to SOD1^G93A^ Tg mice (Fig. 5e–h). Interestingly, these data imply that IL-19 affects expression levels of both neurotoxic and neuroprotective factors in lumbar spinal cord of SOD1^G93A^ Tg mice.

### Ablation of IL-19 increases TNF-α release from microglia and astrocytes, thereby promoting GDNF production in astrocytes, in SOD1^G93A^ Tg mice

Because IL-19 deficiency increased the expression of TNF-α and GDNF in lumbar spinal cord of SOD1^G93A^ Tg mice (Fig. 5d, f), and in light of a previous observation that TNF-α induces GDNF release from astrocytes [25], we investigated the interaction between TNF-α and GDNF in microglia and astrocytes from ALS mice.

**Fig. 6:**
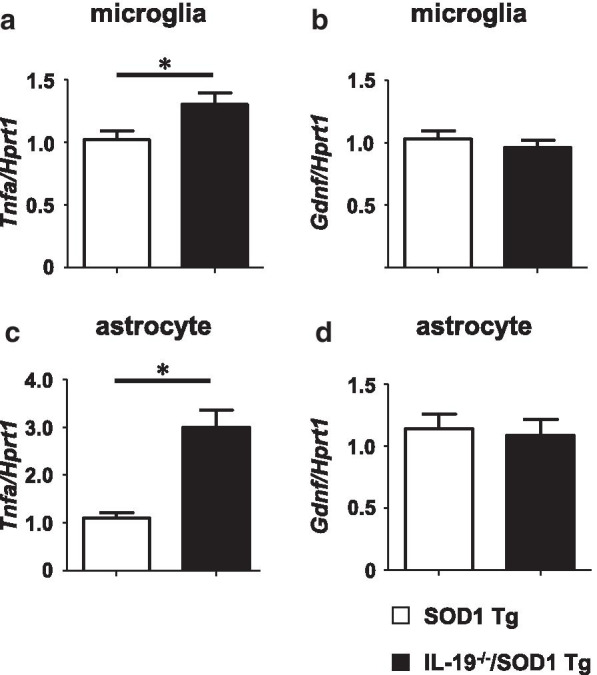
Ablation of IL-19 upregulates TNF-α expression in primary microglia and astrocytes from SOD1^G93A^ Tg. **a**, **b** qPCR data for TNF-α and GDNF expression in LPS (1000 ng/ml)-stimulated primary microglia from SOD1^G93A^ Tg and IL-19^−/−^/SOD1^G93A^ Tg. **c**, **d** qPCR data for TNF-α and GDNF expression in LPS (1000 ng/ml)-stimulated primary astrocytes from SOD1^G93A^ Tg and IL-19^−/−^/SOD1^G93A^ Tg. Values are means ± SD (n = 4). *p < 0.05

First, we sought to determine whether IL-19 deficiency would alter the expression levels of TNF-α and GDNF in microglia and astrocytes from ALS mice. We used LPS to stimulate glial cell cultures because previous studies revealed that mutant SOD1 induces microglial activation via TLR4 and CD14, which serve as the receptor complex for LPS [26, 27]. TNF-α expression was higher in 24-h LPS-stimulated primary microglia or astrocytes from IL-19^−/−^/SOD1^G93A^ Tg mice than in those from SOD1^G93A^ Tg mice (Fig. 6a, c), whereas levels of GDNF were not affected (Fig. 6b, d).

**Fig. 7:**
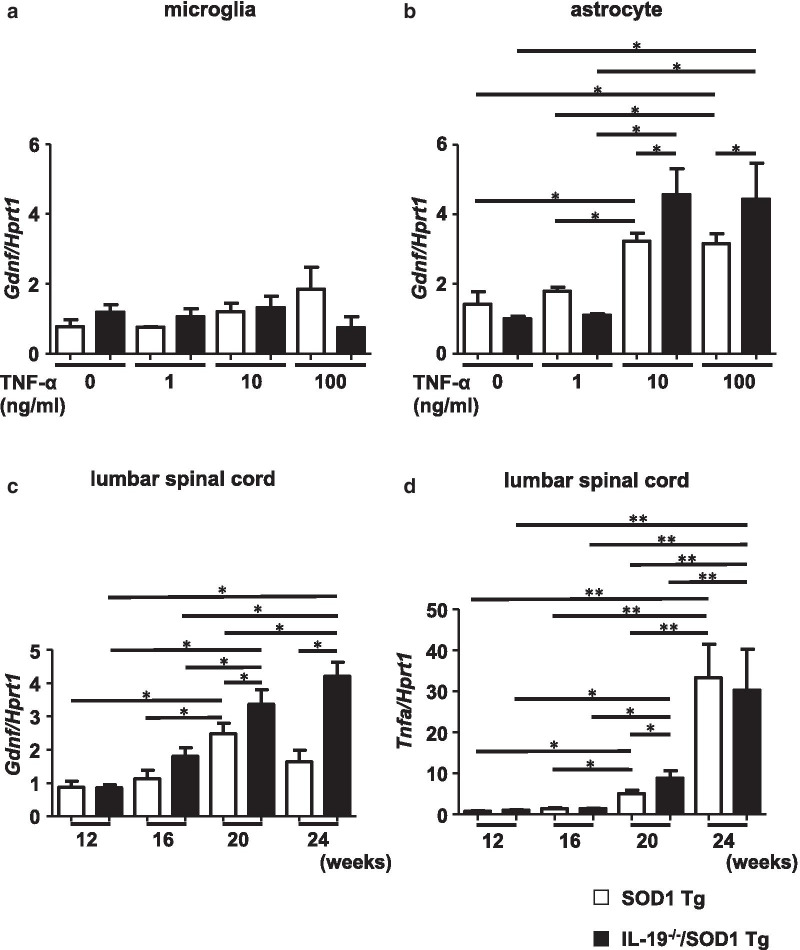
Ablation of IL-19 enhanced TNF-α–induced upregulation of GDNF in primary astrocytes and lumbar spinal cord from SOD1^G93A^ Tg mice. **a** qPCR for GDNF mRNA in primary microglia stimulated with TNF-α (0–100 ng/ml). Values are means ± SD (n = 4). *p < 0.05. **b** qPCR for GDNF mRNA in primary astrocytes stimulated by TNF-α (0–100 ng/ml). Values are means ± SD (n = 4). *p < 0.05. **c** qPCR data for GDNF expression in lumbar spinal cord from SOD1^G93A^ Tg and IL-19^−/−^/SOD1^G93A^ Tg. Values are means ± SD (n = 6). *p < 0.05. **d** qPCR data for TNF-α expression in lumbar spinal cord from SOD1^G93A^ Tg and IL-19^−/−^/SOD1^G93A^ Tg. Values are means ± SD (n = 6). *p < 0.05 when data of 12–20-week-old mice were analyzed. **p < 0.05 when data of 12–24-week-old mice were analyzed

Next, we investigated whether IL-19 deficiency alters the expression level of TNF-α–induced GDNF in microglia and astrocytes from ALS mice. For this purpose, we stimulated primary microglia and astrocytes from SOD1^G93A^Tg and IL-19^−/−^/SOD1^G93A^Tg mice with TNF-α (0–100 ng/ml) for 24 h. TNF-α stimulation dose-dependently upregulated GDNF expression level in astrocytes (Fig. 7b), but not in microglia (Fig. 7a). Moreover, primary astrocytes from IL-19^−/−^/SOD1^G93A^ Tg mice exhibited a significantly greater increase in GDNF expression level than those from SOD1^G93A^Tg mice in response to TNF-α stimulation (Fig. 7b). These results suggested that astrocytes secrete GDNF upon stimulation with TNF-α released from microglia and astrocytes, which acts in an autocrine and paracrine manner.

In addition, we examined the levels of TNF-α and GDNF in the lumbar spinal cord over a time course. GDNF expression was significantly higher in IL-19^−/−^/SOD1^G93A^ Tg mice after the late stage (20–24 weeks) than in SOD1^G93A^Tg mice as the TNF-α upregulation (Fig. 7c, d). The chronological expression data in lumbar spinal cord are consistent with our in vitro observations of astrocytic release of GDNF in response to TNF-α stimulation.

## Discussion

Previous studies showed that IL-19 acts in an autocrine and paracrine negative-feedback regulator to limit pro-inflammatory responses by microglia and macrophages [9, 10], and that it can slow the progression of microglia/macrophage-mediated CNS disorders such as stroke [14] and spinal cord injury [15]. In fact, we recently showed that IL-19 deficiency worsened microglia/macrophage-mediated experimental autoimmune encephalitis, whereas IL-19 treatment abrogated this condition by inhibiting macrophage activation [28]. In this study, we observed upregulation of IL-19 expression in lumbar spinal cord of SOD1^G93A^ Tg mice in parallel with disease progression (Fig. 1). Therefore, we hypothesized that ablation of IL-19 would exacerbate disease progression in SOD1^G93A^ Tg mice.

**Fig. 8:**
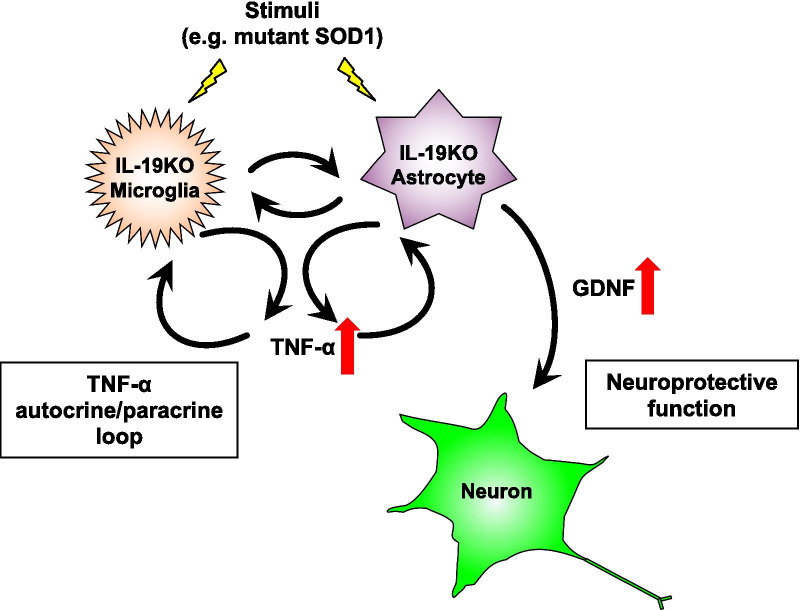
Model of the roles of TNF-α and GDNF in glia-mediated neuroprotection in ALS. Stimuli such as mutant SOD1 induce microglia and astrocytes to release TNF-α. Secreted TNF-α induces GDNF release from astrocytes in an autocrine and paracrine manner, resulting in enhancement of neuroprotection. Blockade of IL-19 signaling may augment this TNF-α–induced GDNF neuroprotective cascade (red arrows)

Unexpectedly, however, we observed that ablation of IL-19 improved motor function at the late stage in SOD1^G93A^ Tg mice (Fig. 2). At this stage, immunohistochemical analyses detected that IL-19 deficiency resulted in conversion of microglia from a pro-inflammatory and neurotoxic phenotype to an anti-inflammatory and neuroprotective phenotype (Fig. 4). qPCR analyses revealed that expression levels of TNF-α and GDNF in lumbar spinal cord were higher in IL-19^−/−^/SOD1^G93A^ Tg mice than in SOD1^G93A^ Tg mice (Fig. 5). Moreover, IL-19 deficiency enhanced TNF-α upregulation in microglia and astrocytes and augmented TNF-α–induced GDNF upregulation in astrocytes from SOD1^G93A^ Tg mice, which was corroborated by the chronological expression levels of GDNF and TNF-α in the lumbar spinal cord of these mice (Figs. 6 and 7). Due to the technical difficulties of isolating and maintaining glial cells from diseased adult mice, we used primary glial cell cultures from neonatal mouse brains to represent disease phenotypes in adult mice. In general, cell culture models recapitulated the disease progression of animal models on a compressed time scale. In ALS models, previous studies also demonstrated that neonatal primary cultures from mutant SOD1 transgenic mice underwent significant phenotypic changes corresponding to those in adult mice [29–31]. Therefore, we believe that our cell models at least partly recapitulated the disease pathology in ALS mice. Interestingly, previous studies documented that TNF-α promotes TNF-α release from microglia and astrocytes in an autocrine and paracrine manner, mediated by TNF-α receptor 1 (TNFR1) [25, 32–34], and that TNF-α induces astrocytic GDNF production [25]. Other studies reported TNF-α elevation in the spinal cord of SOD1^G93A^ Tg mice prior to disease onset, and showed that ablation of TNFR1 but not TNFR2 enhanced motor neuron loss and accelerated disease progression in SOD1^G93A^Tg mice [35, 36]. Microglia-derived TNF-α plays a crucial role as a paracrine signal to regulate astrocytic neuroprotective functions [37], and microglia in SOD1^G93A^ Tg mice produce high levels of TNF-α [26]. Based on these findings, we hypothesized that the TNF-α–GDNF axis would exert neuroprotective effects in ALS pathogenesis (Fig. 8). Mutant SOD1–derived pro-inflammatory stimuli induce microglia and astrocytes to release TNF-α in an autocrine and paracrine manner. Secreted TNF-α induces astrocytic GDNF release, which exerts a neuroprotective effect. Ablation of IL-19 promotes neuroprotection by enhancing this TNF-α–GDNF neuroprotective cascade.

Several lines of evidence indicate that activated microglia and astrocytes are pivotal players in ALS pathogenesis, and that glial activation exerts both neurotoxic and neuroprotective effects depending on its spatiotemporal distribution [29, 38]. In particular, glial activation by cytokines often shows conflicting abilities in neurotoxicity and neuroprotection [39]. For example, overexpression of TGF-β1, an anti-inflammatory cytokine, paradoxically accelerates the disease progression of SOD1^G93A^ Tg by astrocytic activation [40]. Furthermore, IL-10, a major anti-inflammatory cytokine, exacerbates amyloid-β plaque burden and cognitive impairment, whereas IL-10 deficiency enhanced amyloid-β clearance in Alzheimer’s disease model mice [41, 42]. We also observed that the level of IL-1β in the lumbar spinal cord was higher in IL-19^−/−^/SOD1^G93A^ Tg mice than in SOD1^G93A^ Tg mice. IL-1β is a major pro-inflammatory cytokine that enhances glial NF-κB activation and subsequent TNF-α production [43]. Therefore, it is possible that IL-1β elevation could induce and augment glial TNF-α production in IL-19^−/−^/SOD1^G93A^ Tg mice.

IL-19 also exerts a potent anti-inflammatory effect on CNS-infiltrating monocytes and macrophages under Th1-prone condition [14, 15], whereas it exerts a pro-inflammatory effect on tissue-infiltrating monocytes/macrophages and fibroblasts in Th2-prone conditions [16–18, 44]. Previous studies also reported conflicting observations of IL-19 effects (pro-inflammatory *vs.* anti-inflammatory) even in similar mouse models of colitis [10, 45], suggesting that the effects of IL-19 depend strongly on the condition and distribution of inflammation. In addition to the unexpected pro-inflammatory effect of IL-19 in ALS mice, we observed another small discrepancy in the trends of GDNF and TNF-α expression levels in lumbar spinal cord in the end stage (24 weeks) (Fig. 7c, d). Although differential expression levels of TNF-α were observed between SOD1^G93A^ Tg and IL-19^−/−^/SOD1^G93A^ Tg mice in the late stage (20 weeks), rapidly increasing TNF-α expression in the end stage reached similar levels in both Tg mice (Fig. 7d). By contrast, GDNF expression was significantly higher in IL-19^−/−^/SOD1^G93A^ Tg mice than in SOD1^G93A^ Tg mice from the late to the end stage (Fig. 7c). Nevertheless, these mice had similar lifespans. Strong upregulation of TNF-α in the end stage may have exacerbated neuroinflammation, disrupted CNS homeostasis, and overwhelmed the neuroprotective effect of GDNF. Further investigations are needed to elucidate these issues.

Our observations reveal a complex neuro–glia–immune interaction under inflamed conditions in ALS mice. Spatiotemporal manipulation of glial activation might facilitate the development of novel therapeutic strategies for neurodegenerative diseases such as ALS.

## Conclusions

In this study, we demonstrated that IL-19 deficiency delayed disease onset and improved motor function at the late stage in the SOD1^G93A^Tg mice. Ablation of IL-19 slowed the disease progression of ALS mice by enhancing glial neuroprotection through such glia-secreted factors as TNF-α and GDNF. Our results suggest that blockade of IL-19 signaling represents a potential therapeutic strategy for ameliorating neurodegenerative diseases including ALS.

## Data Availability

The datasets used and analyzed during this study are available from the corresponding author on reasonable request.

## Abbreviations

ALS: Amyotrophic lateral sclerosis
BDNF: Brain-derived neurotrophic factor
CNS: Central nervous system
DMEM: Dulbecco’s modified Eagle’s minimum essential medium
FBS: Fetal bovine serum
GDNF: Glial cell–derived neurotrophic factor
GFAP: Glial fibrillary acidic protein
Hprt1: Hypoxanthine phosphoribosyltransferase 1
IL-19: Interleukin-19
Iba1: Ionized calcium-binding adapter molecule 1
IL-1β: Interleukin-1 beta
SOD1: Superoxide dismutase 1
IL-20Rα: IL-20 receptor alpha subunit
IL-20Rβ: IL-20 receptor beta subunit
LPS: Lipopolysaccharide
iNOS: Inducible nitric oxide synthase
PBS: Phosphate-buffered saline
qPCR: Quantitative reverse transcription PCR
TNF-α: Tumor necrosis factor alpha
TGF-β1: Transforming growth factor beta 1
